# Accumulation of antimony and lead in leaves and needles of trees: The role of traffic emissions

**DOI:** 10.1016/j.heliyon.2023.e13548

**Published:** 2023-02-08

**Authors:** Håkan Pleijel, Jenny Klingberg, Bo Strandberg, Henrik Sjöman, Göran Wallin

**Affiliations:** a University of Gothenburg, Biological and Environmental Sciences, P.O. Box 461, SE-40530, Gothenburg, Sweden; b Gothenburg Botanical Garden, Carl Skottsbergs gata 22A, SE-41319, Gothenburg, Sweden; c Gothenburg Global Biodiversity Centre, Carl Skottsbergs gata 22B, SE-41319, Gothenburg, Sweden; d Lund University, Division of Occupational and Environmental Medicine, SE-22100, Lund, Sweden; e Department of Occupational and Environmental Medicine, Region Skåne, SE-22381 Lund, Sweden; f Swedish University of Agricultural Science, Department of Landscape Architecture, Planning and Management, 23053 Alnarp, Sweden; g Environmental Change Institute, School of Geography and the Environment, University of Oxford, South Parks Road, Oxford, OX1 3QY, United Kingdom

**Keywords:** Accumulation, Gothenburg, Leaf age, Needle age, PAH, Pb, Sb, Traffic

## Abstract

Antimony (Sb) is a toxic metalloid, which has been increasingly used in the brake lining of vehicles, and increased concentrations have been found in soils near abundant traffic. However, since very few investigations of Sb accumulation in urban vegetation have been undertaken there exists a knowledge gap. We studied the concentrations of Sb in leaves and needles of trees in the Gothenburg City area, Sweden. In addition, lead (Pb), also associated with traffic, was investigated. Sb and Pb concentrations of *Quercus palustris* leaves at seven sites with contrasting traffic intensity varied substantially, correlated with the traffic-related PAH (polycyclic aromatic hydrocarbon) air pollution at the sites and increased during the growing season. Sb but not Pb concentrations were significantly higher in needles of *Picea abies* and *Pinus sylvestris* near major roads compared to sites at larger distances. In *Pinus nigra* needles at two urban streets both Sb and Pb were higher compared to an urban nature park environment, emphasising the role of traffic emissions for these elements. A continued accumulation of Sb and Pb in three years old needles of *Pinus nigra*, two years old needles of *Pinus sylvestris* and eleven years old needles of *Picea abies* was observed. Our data suggest a pronounced link between traffic pollution and Sb accumulation in leaves and needles, where the particles carrying Sb seem not to be transported very far from the source. We also conclude that there exists a strong potential for Sb and Pb bioaccumulation over time in leaves and needles. Implications of these findings are that increased concentrations of toxic Sb and Pb are likely to prevail in environments with high traffic intensity and that Sb can enter the ecological food chain by accumulation in leaves and needles, which is important for the biogeochemical cycling.

## Highlights

• Leaf antimony (Sb) and lead (Pb) concentrations were significantly increased near traffic.
• Leaf concentrations of Sb and Pb correlated with the atmospheric PAH concentration.
• Leaf Sb concentration declined with increasing distance from large roads.
• Older plant tissue had consistently higher Sb and Pb concentrations than younger.

## 1 Introduction

Antimony (Sb) is a toxic and potentially carcinogenic metalloid [[1], [2], [3]], the use of which has increased strongly in some sectors of the society during the last decades. Environmental contamination has long been known to exist in the vicinity of Sb mining sites [4]. This element is also used in fire retardants, shooting munition and electronic devices [[5], [6], [7]]. In addition, Sb (as Sb_2_S_3_) has been increasingly used in the brake lining of vehicles, replacing asbestos since the 1980s [8,9], resulting in non-exhaust vehicle emissions. Further, the authors of Jiang et al. [2], in their global analysis of observations of atmospheric Sb concentrations, found that Sb concentration were higher in urban and suburban environments compared to background sites.

Consequently, material wear has led to a spread of this element in the environment, as exemplified by the increased concentrations in soils nearby major roads in the Cologne region, Germany [10] and the increased Sb concentration in airborne dust at a site with high traffic intensity compared to that of a low traffic site in Munich, Germany [11]. Chang et al. [12] investigated the distribution of Sb in street dust of 19 cities in China. They found concentrations in cities to be increased compared to background sites and that concentrations were significantly higher in transportation areas compared to industrial and residential areas.

Using observations of the concentration of a range of metal(loid)s in road tunnels in Gothenburg, Sweden, it was found that the increase of the Sb concentration was larger than that of any other investigated element and concluded that it was derived from brake wear [13]. Further, Bukowiecki et al. [14], studying real-world emission factors for Sb from brake wear in Switzerland, observed that Sb was predominantly found in the fraction of particles with aerodynamic diameters in the range 2.5–10 μm, which agrees with the observations by Földi et al. [10] that the Sb soil contamination declines quickly with increasing distance from the verge of the road. However, in an analysis of a global database of atmospheric Sb concentrations, the mass fraction of Sb was found to be higher in the particle fraction PM_2.5_ (particles with an aerodynamic diameter < 2.5 μm) compared to PM_10_ (particles with and aerodynamic diameter < 10 μm) [2]. Sternbeck et al. [13] also found an increase in Pb concentrations in their tunnel measurements, although smaller than for Sb. The authors attributed it to wear of car components, rather than combustion, like for Sb. [9] state that brake linings also can contain significant amounts of Pb. Consequently, lead may continue to be a pollution metal affected by non-exhaust traffic emissions, although the large and widespread problem of emissions of Pb as an additive to petrol has declined strongly as shown e.g. in Europe-wide moss (bryophyte) inventories [15].

Several crops and other plant types have been shown to accumulate high concentrations of Sb in mining areas (e.g., Feng et al. [16]), although with substantial variation in uptake and Sb tolerance between different species or genotypes [17]. It is also established that metals and metalloids can be taken up by plants both through roots and by foliar uptake [6,18], and that Sb uptake by terrestrial plants is proportional to the environmental concentration over a wide range of concentrations [18]. With respect to Sb contamination of urban vegetation available literature is very limited. However, Parviainen et al. [19] showed, based on an extensive study using lichens as bioindicators in Granada, Spain, Sb to be an efficient tracer of non-exhaust emissions in urban air pollution. The authors of Dietl et al. [11] used standardized grass cultures to investigate the accumulation of Sb in two locations differing in traffic load and found significantly higher concentrations in grass at the site with higher traffic intensity. The accumulation of Sb in urban trees has not been investigated to any large extent, although [20] made observations of a range of elements, including Sb and Pb, in leaves and needles of trees in and near Barcelona, Spain. Hegrová et al. [21] studied the contamination by a range of elements of near-road environments in the Czech Republic, in soil and Scots pine (*Pinus sylvestris*) needles. They found the concentrations of Sb to be the most strongly increased out of 19 elements. Pb also showed a substantial increase in roadside Scots pine needles compared to a control area, but the increase in the Pb concentration was smaller than for Sb in that investigation.

It has been observed earlier that Sb can bioaccumulate over time in plant materials. Wyttenbach et al. [22] found the Sb concentration of Norway spruce (*Picea abies*) needles to increase monotonically over time in current year (C) needles and 1-year old (C+1) needles. In addition, Wyttenbach et al. [23] observed Sb to accumulate over the range of five needle age classes in another study with Norway spruce. An accumulation of Pb over consecutive needle age classes in conifers has also been observed (e.g., Robarge et al. [24]). Such an accumulation process is relevant to the use of leaves or needles as passive samplers of Sb and Pb in biomonitoring of the urban environment. If the concentration of Sb or Pb increases from year to year in conifer needles it is essential that sampling in biomonitoring is standardized with respect to needle age, and older needles may provide a stronger chemical signal [25]. Sevik et al. [26] investigated seven woody species as potential biomonitors for Pb and found that the species differences were rather small with respect to Pb accumulation. Juranović Cindrić et al. [27] studied four Pinus species as potential bioindicators for metals, including Pb. These authors, however, found relatively large variation in metal accumulation among species. Similarly, Levei et al. [28] found substantial inter-species differences in metal concentrations in leaves of Populus nigra, e.g., with respect to Pb.

Polycyclic aromatic hydrocarbons (PAHs) are toxicologically important air pollutants. They are formed as a result of incomplete combustions of organic material, for example in vehicle engines. Thus, traffic exhausts are a major emission source of PAHs, dominating in urban environments with high traffic density [29,30]. Strong contrasts have been found between differently traffic polluted parts of urban areas with respect to PAH concentrations of the air [31,32]. The concentration of PAH is a powerful indicator of local traffic pollution in a city [29,33], which can be used to investigate the connection between exhaust pollution and non-exhaust pollution represented by e.g., Sb and Pb.

There are clear indications in the literature of a substantial contamination of non-exhaust Sb and Pb in areas with abundant traffic. However, very little information on the accumulation of these elements in the foliage of trees in these environments has been published in the scientific literature. It is therefore strongly motivated to investigate the Sb and Pb concentrations in leaves and needles of trees in environments affected by traffic pollution. Furthermore, to assess the accumulation of Sb and Pb in needles and leaves as a path into the ecological food web, information on the changes in concentration of these elements over the complete lifetime of leaves and needles is essential. This has not been investigated to any large extent. To address these knowledge gaps, we sampled trees within and near the conurbation of the City of Gothenburg, south-west Sweden, at sites with different levels of traffic pollution and in leaf and needle tissues with different ages. Our hypotheses were:

• Sb and Pb concentrations of tree leaves and needles are higher in more traffic polluted environments.
• Sb and Pb concentrations increase with leaf or needle ageing in broadleaved trees and conifers.

In addition, we discuss the potential role of leaves and needles as biomonitors for Sb and Pb.

## 2 Materials and methods

### 2.1 Investigated species and sites

To address the hypotheses, we sampled leaves and needles in four different campaigns where the effects of traffic on Sb and Pb concentrations could be assessed. Thus, to investigate the relationship between traffic pollution and leaf or needle Sb and Pb concentrations (first hypothesis) we investigated four tree species growing in city environments with contrasting level of traffic pollution (two campaigns) or at different distances from major roads (two campaigns). In the city campaigns both a broadleaved tree and a conifer were included to be able to reflect a possible influence of plant functional type. In all cases plant leaves or needles of different ages were sampled to study the accumulation Sb and Pb over time (second hypothesis). To further investigate the second hypothesis, the Sb and Pb concentrations of the full range of needle age classes in one conifer was included in the study.

Observations were made of the following species and sites (coordinates and general characteristics of the sites are given in Table 1 with further details provided in Table S1 of the supplementary information; location of sampling sites also shown in the map in Fig. S1). Leaf samples of pin oak (*Quercus palustris*) were taken on 26–28 June and 19–20 September 2018 at seven sites in the city of Gothenburg. These sites represent a wide range of traffic-related pollution loads, from the most traffic polluted areas in the city center to less polluted suburban environments on the periphery of the city. Black pine (*Pinus nigra*) was sampled at three sites in the city of Gothenburg on 26–28 June 2018. Two of these were identical with pin oak sampling sites situated in city street environments which were contrasted with the third site in the arboretum of Gothenburg Botanical Garden located in a nature reserve surrounded by the city but at considerable distance (500 m) from the closest road. The needle age classes C+1 (the needle cohort from last year, current plus one year old) and C+3 (current plus three years old) were sampled. Scots pine (*Pinus sylvestris*) was sampled at two distances (10 m and 40 m) from a relatively busy traffic route (ca 13,000 vehicles per day) near the Gothenburg conurbation on 19 August 2019. Needle age classes C and C+2 were sampled. Norway spruce (*Picea abies*) samples were taken in two different settings: 1) On 20 August 2019 for C and C+2 needles at four distances (1, 10, 40, 80 m) from the edge of a forest stand situated ca 35 m from a busy motorway (ca 50,0000 vehicles per day) north of Gothenburg; 2) On 22 September 2020 in the Änggårdsbergen nature reserve next to the Gothenburg Botanical Garden. Here, the full range of needle age classes present on the trees were sampled, eleven in two trees and nine in one tree, to study the capacity for long-term bioaccumulation of Sb and Pb in conifer needles.

**Table 1:** Site ID, location, expected pollution level, if air PAH measurements were made, investigated species (Qp, *Quercus palustris*; Pn, *Pinus nigra*; Ps, *Pinus sylvestris*; Pa, *Picea abies*) and short description of the study sites. X indicates which tree species were sampled at each site with respect to Sb and Pb analysis, and if atmospheric PAH was monitored at that site. C, current year needles, C+1, one year-old needles etc. Further details concerning the sampling sites are presented in Table S1 of the supplementary information.

Site ID	Coordinates		Expected pollution level	PAH	Qp	Pn	Ps	Pa	Description
NET	57.71194 N	11.97278 E	Very high	X	X				Central station in the center of town: *Nils Ericson terminalen* leaves sampled June and Sept. 2018
MÖL	57.68917 N	11.99500 E	High	X	X	X			Urban street: *Mölndalsvägen* leaves sampled June and Sept., C+1 and C+3 needles June 2018
FRI	57.71889 N	11.96222 E	High	X	X				Urban traffic route: *Frihamnen* leaves sampled June and Sept. 2018
KVI	57.72333 N	11.95056 E	High	X	X	X			Urban park: *Kvillebäcksparken* leaves sampled June and Sept., C+1 and C+3 needles June 2018
AKK	57.75194 N	11.98528 E	Moderate	X	X				Suburban residential area: *Akkas gata* leaves sampled June and Sept. 2018
KUN	57.68389 N	11.92694 E	Moderate	X	X				Urban residential street: *Kungsladugårdsgatan* leaves sampled June and Sept. 2018
ANG	57.79528 N	12.05750 E	Low	X	X				Peri-urban park: *Angereds stadspark* leaves sampled June and Sept. 2018
ARB	57.67457 N	11.95522 E	Low	X		X			*Gothenburg Botanical Garden arboretum* C+1 and C+3 needles June 2018
ELL	57.82887 N	12.00344 E	Very high	X				X	Motorway north of Gothenburg City: *Ellesbo* C and C+2 needles sampled August 2019
HIS	57.76500 N	11.88444 E	High	X			X		Traffic route north-west of Gothenburg City: *Hisingsleden* C and C+2 needles sampled August 2019
ÄNR	57.66 N	11.96 E	Low					X	Nature reserve: *Änggårdsbergen nature reserve* All needle age classes from C to C+10 sampled September 2020

At each site included in the study, three trees were sampled. The sample for each tree consisted of leaves or needles from at least three branches. The age of needles in conifers is in most cases very easy to assess, since the needles of different age classes are attached to separate, distinct segments of the branches. Fig. S2 of the supplementary information shows an example of how different needle age classes are separated in evergreen conifers.

### 2.2 Sampling, preparation and analysis of samples

Branches of mature trees which were light-exposed (exception site ELL, which was a dense Norway spruce stand) with leaves or needles in the outer part of the crown were selected. The samples were packed in polyethylene plastic bags, transported in a cool bag, and stored in a freezer (<-18 °C) until further handling. Samples were carefully collected to avoid contamination. Needles were separated from the branch segments at the laboratory. Plant samples were dried at 70 °C in aluminium envelopes until constant weight (at least 48 h) using well ventilated ovens. Thereafter the samples were ground to a fine powder using a ball mill (model MM 301, Retsch, Haan, Germany) equipped with grinding jars and balls made of wolfram carbide. Samples were analysed for the content of 37 elements including Sb and Pb using inductively coupled plasma mass spectrometry (Dry vegetation ICP-MS, 37 elements after digestion in HNO_3_ and then aqua regia: Method Vegetation Analysis VG101 by Bureau Veritas Mineral laboratories, Vancouver, BC, Canada [34]). The laboratory has implemented a quality management system meeting the requirements of ISO/IEC 17025 and ISO 9001. In this paper, Sb and Pb data are presented. The method detection limit (MDL) was 0.02 μg g^−1^ for Sb and 0.01 μg g^−1^ for Pb (ICP-MS). The certified reference material was flour. Some samples, especially in younger plant tissues at cleaner sites, were below the MDL for Sb. If two or all three replicates were below MDL they were excluded from further analyses and are reported as <MDL. If only one of the three replicates was <MDL, that value was set to 0.5 × MDL and was included in the data analysis.

### 2.3 Monitoring of atmospheric PAH concentrations

PAH air concentrations were measured at 2.5 m above ground using passive sampling technique. The samplers were exposed approximately one month. See the supplementary information for further details of the measurement periods start and stop time. PUF (polyurethane foam) disk samplers (14 cm diameter, 1.2 cm thickness, surface area 360 cm^2^, density 0.035 g cm^−3^, Klaus Ziemer GmbH, Langerwehe, Germany), used for air PAH sampling, were housed in two stainless steel domes (Tisch Environmental, Inc., Cleves, OH, USA). This sampler design has been calibrated for a number of PAHs, both gaseous and particulate-associated compounds [35,36]. Prior to deployment, the PUF disks were precleaned by Soxhlet extraction for 24 h using dichloromethane (DCM), dried under vacuum, and stored in multiple layers of solvent-rinsed aluminium foil inside airtight polyethylene zip bags. Field blanks, i.e., samplers unopened during the measurement campaign, were processed in parallel with the samples in the analytical procedure to estimate the residual levels of target compounds. After air sampling, the PUF disks were enveloped in rinsed aluminium foil, placed in air-tight polyethylene zip bags, brought to the laboratory, and stored at −18 °C until extraction.

The retrieved field exposed PUF disks were extracted and cleaned following the procedure used earlier for the PUF [37]. Prior to extraction, the samples were spiked with an internal standard (IS) containing deuterated US EPA 16 PAHs (Dr. Ehrenstorfer, Augsburg, Germany). A recovery standard (RS) containing octachloronaphthalene (Ultra Scientific, North Kingstown, RI, USA) was spiked in each sample during the last step before injection. PAHs were then separated and detected by high-resolution gas chromatography/low-resolution mass spectrometry (HRGC/LRMS). The MS instrument was an Agilent 5975C connected to a 7890A GC (Agilent Technologies, Inc., Santa Clara, CA, USA). The MS was operated in electron impact (EI) ionization mode using the selected ion monitoring (SIM), and the GC column was a non-polar capillary column (30 m × 0.32 mm inner diameter and 0.25 μm film thickness, J&WDB-5, Folsom, CA, USA). The published uptake rates by Bohlin et al. [36] were used to quantify air concentrations of all 15 of the US EPA PAHs except naphthalene. Two-ring PAHs, such as naphthalene, may after a 28-day sampling period be in the curvilinear phase of uptake or have reached saturation in the sampling material [36]. Thus, accurate quantification of this compound could not be made, and was therefore excluded. The alkylated PAHs were quantified using published uptake rates presented by Harner et al. [35] or using the uptake rates for a corresponding, or closely related compound. The concentrations of 32 PAHs were determined (see Table S2 of the supplementary information) and the “total air PAH concentration” is the sum of these PAHs. A detailed description of the chemical analysis as well as an account of quality assurance and quality control of PAH analyses is provided in the supplementary information.

### 2.4 Statistical analysis

One-way analysis of variance (ANOVA) with Tukey HSD as the post hoc test was used to evaluate the differences between sites for C+3 needle Sb and Pb concentration of black pine and for September Sb and Pb concentration of pin oak. A mixed designed ANOVA was used to evaluate differences in Sb and Pb concentration between four different distances from a motorway and two different needle age classes of Norway spruce at site ELL; age was set as the within-subjects variable (repeated measures) with two levels. Tukey HSD was used as post-hoc test of the difference between the four sites. ANOVA was made by using IBM SPSS Statistics software package (version 25, SPSS, Inc., Chicago, IL, USA). Student’s *t*-test (two-tailed) was used to test the difference in Sb and Pb concentration of Scots pine C+2 needles from two different distances from the road at site HIS. The dependence of Sb and Pb concentration on needle age in Norway spruce (site ÄNR), as well as the leaf age dependence of Sb concentration in pin oak, was analysed using linear regression, while non-linear regression was used to describe the relationship between Sb and Pb concentration of pin oak leaves with the atmospheric concentration of PAH and the dependence of Pb concentration on needle age at site ÄNR. The significance of deviations from a hypothetical 1:1 relationship in the comparison of September and June concentrations of *Quercus palustris* leaves was investigated by studying if the confidence limits of the regression included 1. All Sb, Pb and PAH data used are available in the Supplementary information.

## 3 Results

### 3.1 Leaf concentrations of Sb and Pb of pin oak at seven sites in the City of Gothenburg

**Fig. 1:**
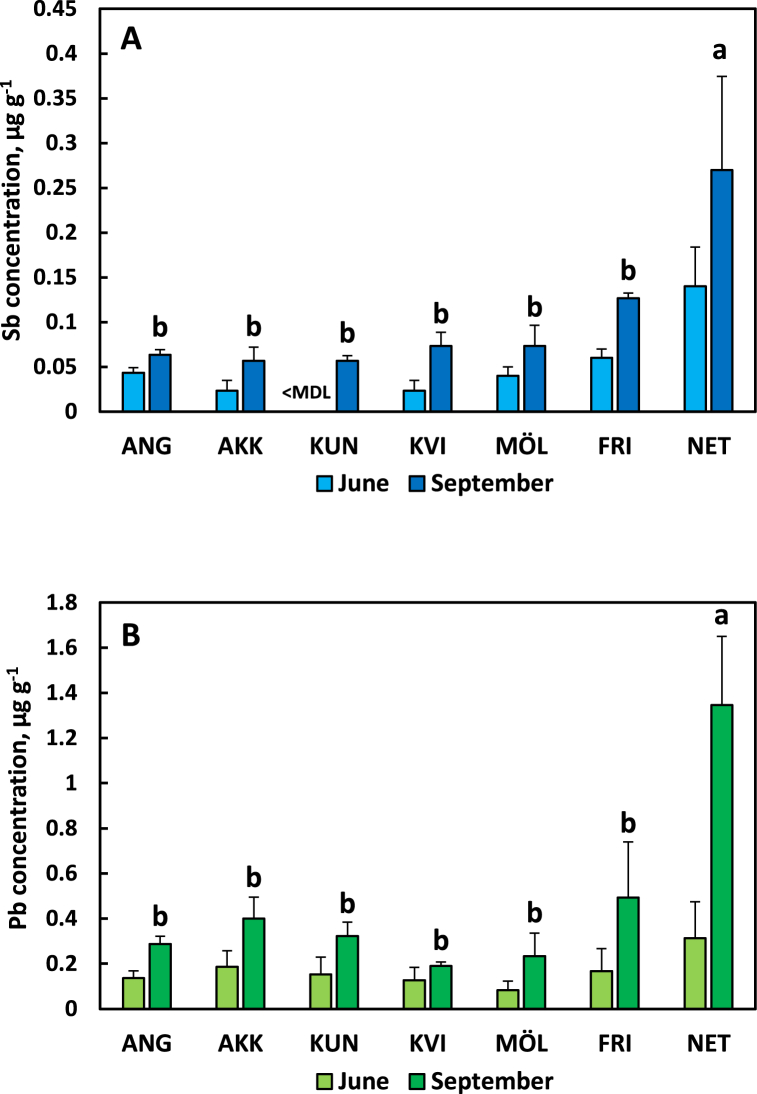
Leaf concentrations of antimony (Sb, panel A) and lead (Pb, panel B) in pin oak (*Quercus palustris*) at seven different sites in the urban landscape of the City of Gothenburg sampled in June and September 2018. <MDL, below method detection limit. Different letters above bars denote statistical difference between sites. Abbreviations of site names are explained in Table 1. Error bars show standard deviation.

The Sb concentration of pin oak leaves increased substantially from June to September and differed between the seven sites of the urban landscape of the City of Gothenburg (Fig. 1A). A similar pattern was observed for Pb (Fig. 1B). June values of Sb for one site was <MDL. Thus, ANOVA was made only for September values and showed that leaf concentrations of Sb and Pb were significantly higher at the most polluted site compared to the rest of the sites.

**Fig. 2:**
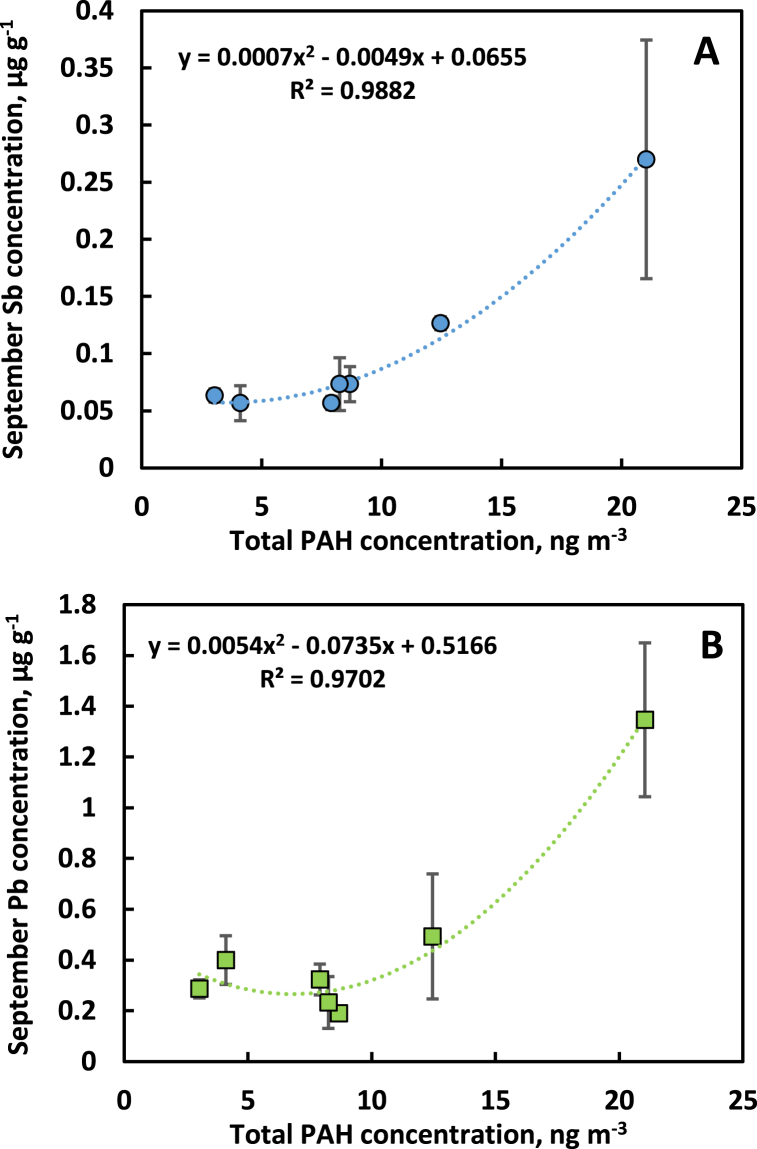
September antimony (Sb, panel A) and lead (Pb, panel B) of pin oak (*Quercus palustris*) leaves vs. the total atmospheric concentration of PAHs (mean of June and September concentrations) at seven sites in Gothenburg with different levels of traffic pollution. Error bars show standard deviation.

There was a strong but non-linear relationship between September leaf Sb concentration in pin oak and the total air PAH concentration during the growing season (represented by the mean of June and September concentrations) of the air at the sites (Fig. 2A). The relationship for Pb was similar (Fig. 2B). The city center site (NET) was clearly highest for the concentrations of Sb, Pb and PAH. In the PAH air concentration range 8–21 ng m^−3^ the relationship was essentially linear (R^2^ = 0.996 for Sb and 0.967 for Pb, data not shown). This could indicate that local Sb and Pb contamination, above a more generally distributed background of Sb and Pb, becomes important only above a certain level of traffic pollution.

**Fig. 3:**
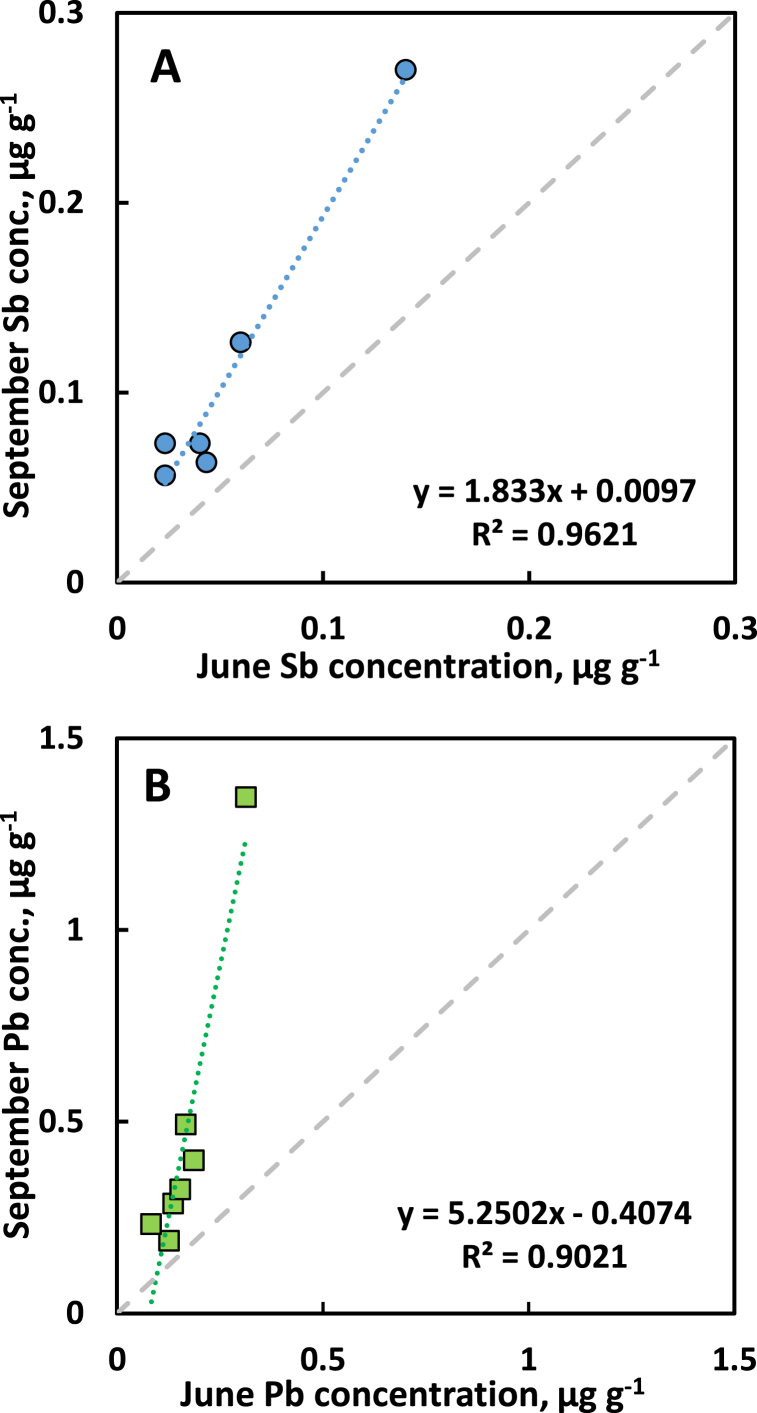
Concentration of antimony (Sb, panel A) and lead (Pb, panel B) in leaves of pin oak (*Quercus palustris*) in September vs. June at sites with contrasting traffic pollution. The broken grey line denotes a hypothetical 1:1 relationship.

Fig. 3A and B shows the relationship between the concentration of Sb and Pb, respectively, of pin oak leaves in September vs. June at sites with contrasting traffic pollution (six data points for Sb and seven for Pb since the Sb concentration was <MDL at one site in June). The pattern is consistent with the slope coefficient of the linear regression suggesting a typical increase in leaf Sb concentration of 83% between June and September. The rate of accumulation of Pb was considerable larger for Pb compared to Sb. For both elements the slope coefficient was significantly larger than unity (*p* < 0.05 for Sb, *p* < 0.01 for Pb).

### 3.2 Needle concentrations of Sb and Pb of black pine at three sites in the City of Gothenburg

**Fig. 4:**
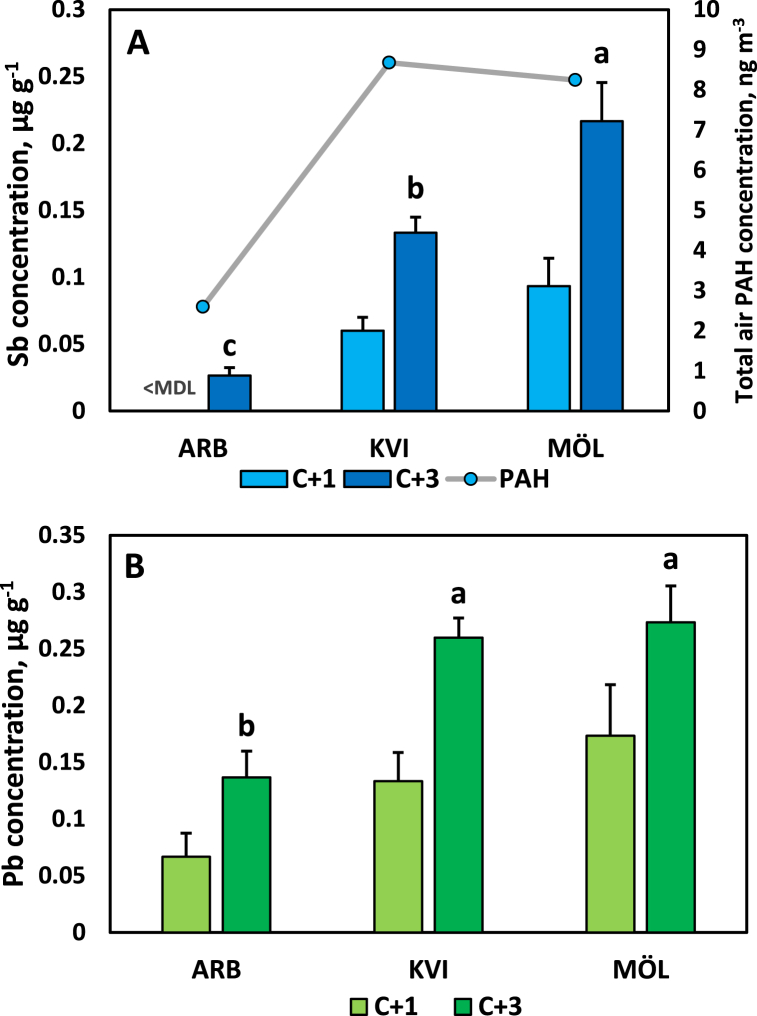
Concentration of antimony (Sb, panel A) and lead (Pb, panel B) in 1-year old (C+1) and 3-year old (C+3) needles of black pine (*Pinus nigra*) in the arboretum (ARB) and two sites (KVI and MÖL) with traffic exposure in the City of Gothenburg. <MDL, below method detection limit. In panel A also the total air PAH concentrations are shown. See Table 1 for details concerning the sampling sites. Different letters above bars denote statistical difference between sites with respect to concentrations for the C+3 needles. Error bars show standard deviation.

Fig. 4A and B compare the Sb and Pb concentrations in C+1 and C+3 needles of black pine at two urban street sites and in the arboretum, ca 500 m away from traffic. Sb values for C+1 needles in the arboretum (site ARB) were <MDL. Thus, C+1 needles were not included in the ANOVA. For C+3 needles, the difference between the sites was statistically significant (*p* < 0.001) according to the ANOVA. The post-hoc test showed that the Sb concentration at the three sites were all different from each other, while for Pb the city street sites differed from the arboretum but not from each other. The Sb concentration was higher in C+3 compared to C+1 needles at all sites. Total air PAH concentrations (Fig. 4A) were clearly higher in the two city street sites, largely but not perfectly in line with the increased needle Pb and Sb concentrations in these sites.

### 3.3 Sb and Pb concentrations of Norway spruce needles in gradient from a motorway

**Fig. 5:**
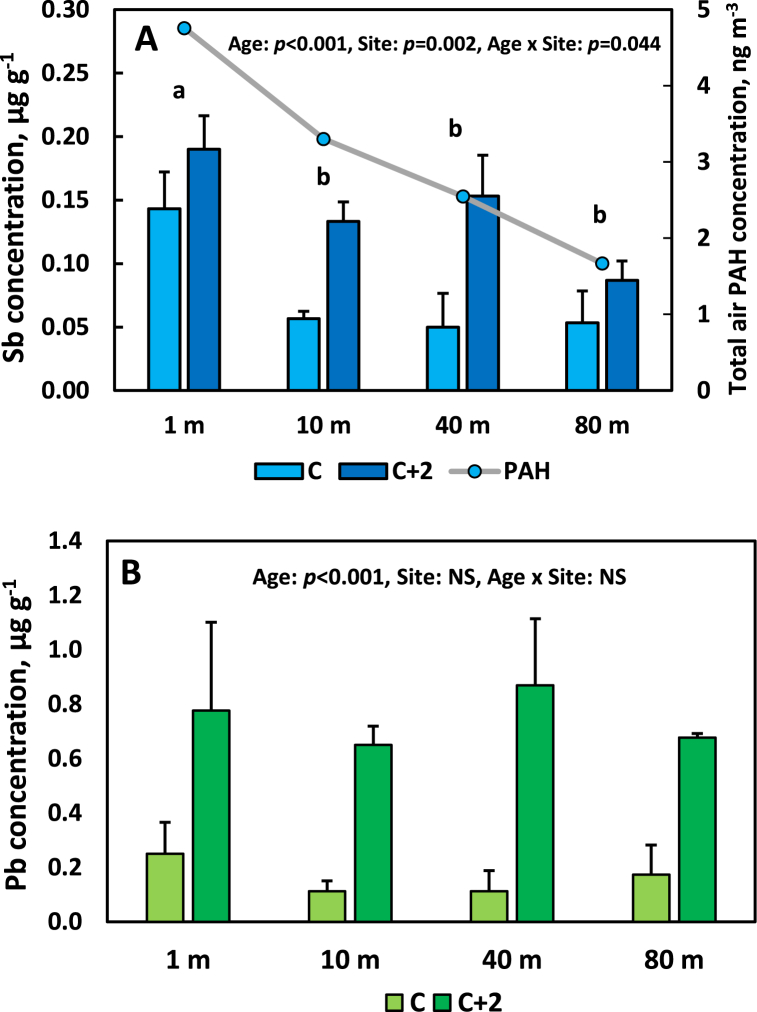
Concentrations of antimony (Sb, panel A) and lead (Pb, panel B) of current (C) and 2-year old (C+2) needles of Norway spruce (*Picea abies*), and total atmospheric PAH concentrations (panel A) at four distances from a forest edge near a motorway at site ELL (see Table 1). Different letters above bars show significant differences between locations. Error bars show standard deviation.

Concentrations of Sb and Pb of C and C+2 needles of Norway spruce in four sites at different distances from a busy motorway is shown in Fig. 5A and B (Site ELL in Table 1). The increase of Sb concentrations was most pronounced for C needles at the site closest to the motorway. The rate of decline in Sb concentration with distance from the road was different for C and C+2 needles, resulting in a significant (*p* = 0.044) interaction between site and needle age, with a steeper decline with increasing distance for C needles compared to C+2. The post-hoc test showed that the Sb concentrations closest to the motorway was significantly different from that at the other distances, which did not differ significantly from each other. In the case of Pb there was no indication of a decline in concentration with increasing distance for C+2 needles, only for C needles, and only the needle age effect was statistically significant according to the ANOVA (p < 0.001). Similar to *Quercus palustris* leaves (Fig. 3), the rate of increase in concentration of Pb with increasing foliage age was higher than for Sb.

The total atmospheric PAH concentrations of the air are presented in Fig. 5A, showing the highest concentrations closest to the motorway, and a declining trend with increasing distance.

### 3.4 Sb and Pb concentrations of Scots pine needles at two distances from a traffic route

**Fig. 6:**
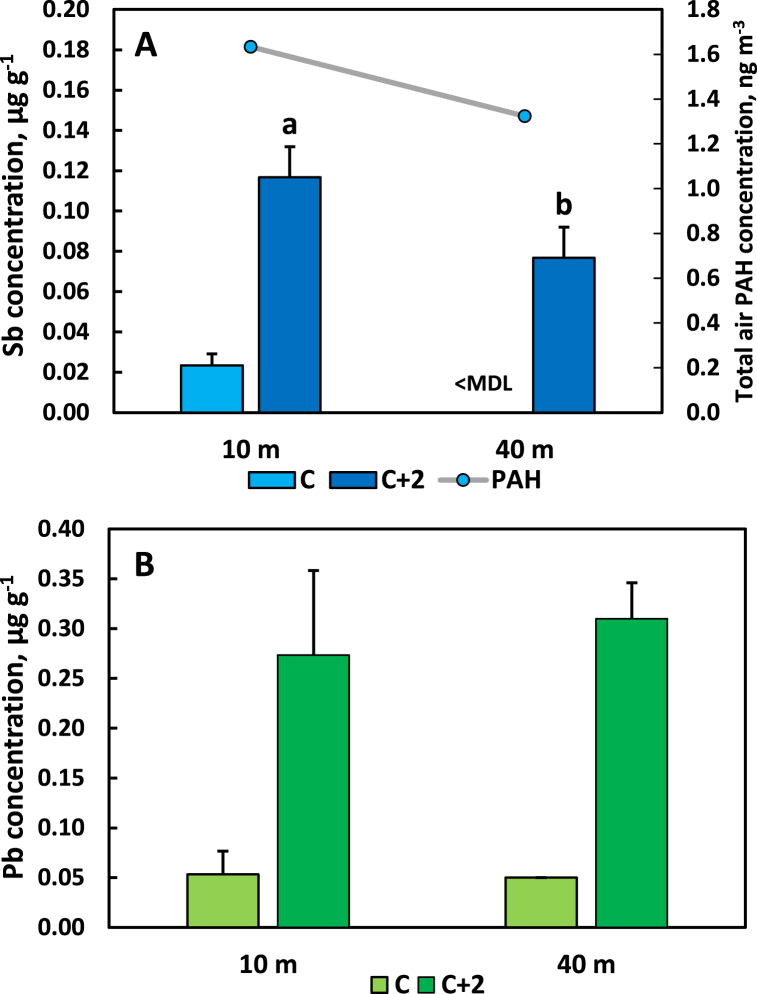
Concentrations of antimony (Sb, panel A) and lead (Pb, panel B) in current (C) and 2-year old (C+2) needles of Scots pine (*Pinus sylvestris*) at two different distances from a traffic route at site HIS (see Table 1). In panel A also the total air PAH concentrations are shown. <MDL, below method detection limit. Different letters above bars show significant differences between the sampling locations. Error bars show standard deviation.

Sb and Pb concentrations of C and C+2 Scots pine needles at two distances from a traffic route (site HIS, see Table 1) are displayed in Fig. 6A and B. For C needles the Sb concentration was above MDL only at the position closest to the road. For C+2 needles, all values were above MDL and the *t*-test revealed that the difference in concentration between the distances (Fig. 6A) was statistically significant for this needle age class (*p* = 0.033). The total atmospheric PAH concentration, also shown in Fig. 6A, at 10 m from the road was 1.6 ng m^−3^ while the concentration was 1.3 ng m^−3^ at 40 m at site HIS. For Pb (Fig. 6B), there was no indication of a declining concentration in any of the needle age classes with increasing distance from the traffic route. There was a strong increase in both the Sb and the Pb concentration from C to C+2 needles.

### 3.5 Sb and Pb concentrations of up to eleven needle age classes of Norway spruce in an urban nature reserve

**Fig. 7:**
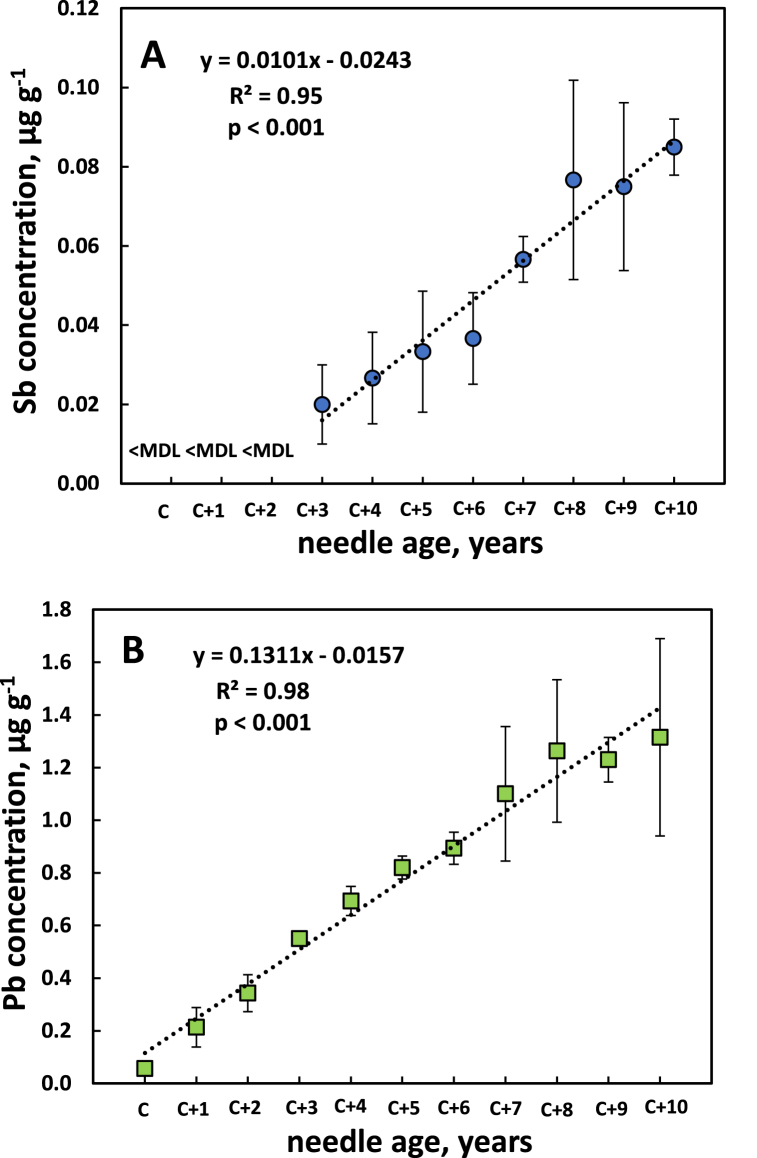
Concentration of antimony (Sb, panel A) and lead (Pb, panel B) in needles of Norway spruce (*Picea abies*) of different ages at site ÄNR (see Table 1), where C denotes current year needles, C+1 1-year old needles etc. <MDL, below method detection limit. Error bars show standard deviation.

The development of the Sb and Pb concentration over different needle age classes of Norway spruce in the Änggårdsbergen nature reserve (site ÄNR), next to the botanical garden, is presented in Fig. 7A and B. For the first three needle age classes values were below MDL for Sb (Fig. 7A). Over the needle age classes C+3 to C+10 there was an essentially linear increase of the Sb concentration, resulting in a statistically significant (*p* < 0.001) relationship. For Pb (Fig. 7B) all values were above MDL and a strong increase in concentration with needle age was observed. This pattern, like that of differently aged needles of black pine and Scots pine, as well as pin oak leaves of different ages, points towards a strong bioaccumulation in foliage with increasing age, in the case of Norway spruce over the full range of needle age classes. However, in the case of Pb, there was a tendency for a levelling off in the rate of increase in concentration for the older needle age classes (Fig. 7B), resulting in a weaker accumulation rate compared to Sb over the full range of needle age classes: the ratio of concentrations C+10 to C+3 (the youngest needle age class with Sb data) was higher for Sb (4.2) compared to Pb (2.4).

## 4 Discussion

Our study found a consistent connection between leaf/needle Sb concentration and the level of traffic intensity as well as with the traffic related PAH air pollution, both between differently polluted areas of the city and at different distances from major roads, although Sb is likely to represent non-exhaust vehicle pollution. The authors of Harrison et al. [29] investigated the relationship between the contamination with a range of metals and concentrations of vehicle exhaust pollutants. They found significant correlations of several elements, including Pb, Cu, Zn and Ba, with the concentrations of nitrogen oxides (exhaust pollutant). That investigation did, however, not include Sb. For Pb we also observed a connection with traffic pollution for the pin oak and black pine data (Fig. 2, Fig. 4B), i.e. between different areas of the city. For Norway spruce at the motorway site a decline in Pb concentration with increasing distance was indicated for C needles, but not for C+2 (Fig. 5B). For Scots pine at the traffic route site outside the city (Fig. 6B) there was no difference in Pb concentration between different distances from the road at all. Thus, differences in Pb concentration between differently polluted areas of the city were more pronounced than local differences at the roads outside the city. Thus, in contrast to Pb at the differently polluted urban sites, and Sb at all traffic polluted sites, the Pb concentrations of tree needles did not change with the distance from roads at the two rural sites. A likely explanation of this pattern is the very extensive legacy Pb deposition to these environments over the long period during which Pb was an important additive to petrol. Since Sb emissions have increased much more recently, this element is likely much less affected by legacy pollution. In the urban environment, with limited soil surfaces, a larger fraction of Pb uptake is likely the result of direct deposition to leaf or needle surfaces. Investigating the soil content of Sb and Pb, not included in the sampling strategy of our investigation, would cast further light on the role of legacy deposition.

In general, the association with traffic pollution, represented by the total atmospheric PAH concentration, was clearer for Sb compared to Pb. Thus, our first hypothesis, that Sb and Pb concentrations of tree leaves and needles are higher in more traffic polluted environments, was supported to a large extent for Sb, and for the sites inside the city also for Pb. An implication of this is that there exists a potential problem with contamination by these two toxic elements of environments influenced by traffic, which needs further investigation with respect to ecotoxic risks and effects on humans.

Our observations of a connection between Sb and traffic were based on several separate observations at different sites. Although very few studies of Sb accumulation in urban vegetation have been published, our results are in general in line with the result by Dietl et al. [11], who found a larger accumulation of Sb in grass exposed in an environment with a higher intensity of traffic compared to a cleaner site. The pattern found in our study also agrees with the observations by Parviainen et al. [19] of a strong connection between traffic intensity and Sb accumulation of epiphytic lichens in Granada, although it should be kept in mind that the uptake of elements by lichens takes place directly from the atmosphere, while trees can take up elements deposited to soil through roots and from deposition directly to leave/needles. Our results also conform with results in the study by Sardans et al. [20], who observed a strong contrast in the concentration of Sb in the moss *Hypnum cupressiforme*, the conifer *Pinus halepensis* and the evergreen broadleaved tree *Quercus ilex* between areas inside and outside of the Barcelona metropolitan area. Thus, although the amount of available information is very limited, existing observations all conform, suggesting the contamination with Sb in urban environments to be a general phenomenon.

Parviainen et al. [19] suggested Sb to be a strong indicator of non-exhaust traffic pollution. This can be explained by the increased use of Sb in braking gear in vehicles [8,9]. It is also in line with the distinct increase in the Sb concentrations in tunnels of Gothenburg [13]. These authors found the increase in Sb concentration to be larger than for the other investigated elements. The increase in Pb concentration, substantial but smaller than for Sb, observed in that study was concluded to be a result of non-exhaust emissions. Further, there is recent evidence that Sb is found in increased concentrations in soil near roads with intensive traffic [10].

Our observations of a relatively steep decline in leaf or needle concentration of Sb with increasing distance from heavy traffic, agree with the suggestion that Sb is mostly found in the coarse particle fraction [14], which to a large extent deposit near the source, thus also in line with the conclusion that soil contamination of traffic related Sb concentrations drops quickly with increasing distance from the road [10]. As mentioned in the Introduction, Jiang et al. [2] found a higher mass fraction of Sb in smaller particles (PM_2.5_) compared to larger (PM_10_) in their extensive global review of atmospheric Sb concentration. However, it should be kept in mind that a substantial part of the non-exhaust particles carrying Sb and Pb were first deposited as road dust, which was later resuspended and deposited in the environment near roads [38]. One should also be aware that emission and deposition of Sb varies with local aspects of traffic flow, such as between road stretches with low or high frequency of braking during traffic jams [19]. In addition, air flow dynamics close to the road is influenced by traffic speed and density, which can affect the distribution of traffic emitted particles.

The investigation of the Sb and Pb concentrations of different needle age classes showed that accumulation continued over several years, in fact over the full range of up to eleven age classes in Norway spruce needles. Even for the other observations regarding conifers, older needles consistently had higher concentrations of Sb and Pb than younger, without exception. Consequently, our second hypothesis, that Sb and Pb concentrations increase with leaf or needle ageing was supported without exception.

This agrees with the observations of [22,23], which showed Sb concentrations to increase monotonically with time in five-year old needles in Norway spruce. Similarly, Robarge et al. [24] found an accumulation of Pb in the three youngest needle age classes in two conifer species in the Appalachian Mountains. These observations highlight the importance of always taking note of the tissue age when sampling leaves and needles for analysis of element content and of not comparing differently aged leaves or needles when assessing the relative level of pollution at different sites in biomonitoring, as pointed out earlier [25,39]. Older leaves, and especially older needles, represent a stronger chemical signal which is an advantage in biomonitoring. Thus, older conifer needles can be recommended for biomonitoring of elements like Sb and Pb, and needle (or leaf) age should always be identified and reported when foliage is used in biomonitoring. Otherwise confounding may occur due to the comparison of differently aged leaves/needles. In many earlier investigations of metal accumulation in conifer needles, only the youngest needle age class(es) have been included. In our study the variation among replicate trees was modest, adding to the usefulness of conifer needles as in biomonitoring of the distribution of Sb and Pb in the urban landscape.

It can be noted that the Sb concentrations of ten-year-old (C+10) needles at the Änggårdsbergen nature reserve (0.085 μg g^−1^), at a substantial distance from the most closely located road, were less than one third of one growing season old pin oak leaves at the most polluted site NET (0.27 μg g^−1^). It was also lower than in C+3 needles of black pine (0.23 μg g^−1^) in city street trees, in C+2 needles of Scots pine trees at a traffic route outside the city (0.12 μg g^−1^), and in C+2 needles of Norway spruce near a motorway (0.19 μg g^−1^). In an earlier study [20] one year-old needles of Aleppo pine (*Pinus halepensis*) were sampled in different parts of the Barcelona conurbation and its surroundings. In the most polluted sites, the Sb concentration was higher (0.43–0.75 μg g^−1^) while in moderately polluted sites Sb concentrations were in the range 0.06–0.14 μg g^−1^, comparable to or lower than in the conifers at the more polluted sites in our study. [20] also investigated the evergreen oak species *Quercus ilex* and found a concentration range of 0.05–0.52 μg g^−1^ for current year leaves. They can be compared with maximum concentrations in *Quercus palustris* leaves in our study was 0.27 μg g^−1^. The higher maximum concentrations in Barcelona can be explained by the fact that the City of Barcelona is larger and more densely populated compared to the City of Gothenburg. It should also be noted that in addition to the number of vehicles, the character of the traffic can influence both the emission levels and distribution of non-exhaust Sb. For example, Sternbeck et al. [13] found indications of heavy-duty vehicles being stronger emitters of Sb than light duty vehicles.

Largely, the distribution of Pb in and near the Barcelona conurbation [20], was similar to that of Sb, with strongly increased concentrations in the city. The highest Pb concentration (1.4 μg g^−1^) in our study was observed for *Quercus palustris* leaves at the most traffic polluted site, which is lower than the observations for current year leaves of *Quercus ilex* in the City of Barcelona (2.8–4.9 μg g^−1^), but higher than values for sites in the surroundings of Barcelona. It can be noted that in our data the Pb concentration of the oldest needle age class (C+10) of *Picea abies* was the same (1.3 μg g^−1^) as in *Quercus palustris* leaves in September at the most polluted site investigated. Thus, it took eleven growing seasons for the *Picea* needles in the urban nature reserve (site ÄNR) to accumulate the same concentration of Pb as the *Quercus* leaves did over one growing season. As mentioned above the concentrations of Sb of the *Quercus palustris* at the most polluted site was three times higher than in the C+10 needles of the urban nature reserve, indicating a stronger connection of Sb pollution with traffic and possibly a larger influence of legacy deposition of Pb over the urban landscape.

In our study leaves and needles were not washed before analysis. Thus, the sampling represents the total content of leaf/needle Sb and Pb, which, as mentioned above, may represent both root uptake and atmospheric deposition [6,18], and the latter may be absorbed by the foliage. There are pros and cons with washing. Sb and Pb on leaf/needle surfaces represent the total deposition (e.g., Hegrová et al. [21]), which is available for consumption by herbivores. On the one hand it is of value to know how much of the leaf/needle Sb that has been integrated with the green tissue. Best would be to analyze both washed and un-washed leaves/needles to maximize relevant information, although it would double the effort and analysis costs. The authors of [40] investigated the effect of intensive washing (three times shaking the leaves for 15 min in distilled water) of *Quercus ilex* leaves sampled in Naples with respect to the concentration of PAH and seven metals (including Pb but not Sb) of the leaves. For metals (but not PAH), concentrations were significantly lower in washed leaves, except Cd, with a larger amount of the metals possible to wash off in more traffic polluted environments. The difference in element concentration between washed and un-washed leaves is likely to vary between climates, e.g., differing in frequency of rainfall and thus natural wash-off of leaves and needles.

A limitation of our study, like several other studies of Sb accumulation in urban vegetation, is that it is based on a relatively limited amount of data representing one city and its surroundings. In addition, this study did not elucidate detailed mechanisms and further field investigations of the deposition and plant uptake of Sb, as well as Pb, will be required to resolve the questions regarding the paths and processes from emission to plant accumulation. This includes identification of the characteristics of the particles with which Sb and Pb are deposited to soil and leaves/needles, the relative importance of plant uptake through roots vs. by foliage as well as the human exposure of these toxic elements and their entrance to the food chains of humans and other organisms to assess the extent of the toxicological risk.

Our study shows that the toxic metalloid Sb concentration is increased in plant material in environments with intensive traffic. This means that there is a higher Sb exposure in this type of environment, which can potentially influence humans and is likely to have increased during the last decades due to the increased use of Sb in brake linings of vehicles. The increased content of Sb in plant material also represents a route for Sb into the food chain [41], which can result in effects in animals and could be a problem in e.g., kitchen gardens, which have been investigated with respect to other toxic elements than Sb (e.g., Warming et al. [42]). Experiments have shown that the Sb concentration of leaves are higher than those of shoots, which in turn were substantially higher than in seeds and storage organs [43]. Since non-exhaust vehicle emissions of Sb have been increasing over the last few decades [8,9] exposure of humans of the toxic element Sb [1,3] should be given further attention in future research. This includes plants grown for food consumption in urban areas.

## 5 Conclusions

The literature on Sb accumulation in the foliage in urban trees is very limited. Our study shows that non-exhaust emissions of Sb can lead to substantially increased concentrations of leaves or needles of trees in a city or near intensive traffic. Sb and Pb concentrations of *Quercus palustris* leaves were 4.8 and 3.8 times higher, respectively, at the most polluted in relation to the least polluted sites investigated in the City of Gothenburg. The leaf concentration of Sb showed strong correlation (R^2^ = 0.99) with the atmospheric PAH concentration representing the level of traffic air pollution. The increase in Sb concentration was indicated to be restricted to areas relatively close to the traffic emission sources. Older plant tissue consistently had higher Sb and Pb concentrations than younger, pointing towards a strong accumulation over time of this element in leaves and needles of trees. For example, three-year old needles of *Pinus nigra* at the most contaminated city site where this species was investigated had 2.3 and 1.6 times higher concentrations of Sb and Pb, respectively, compared to one-year old needles. The accumulation continued in up to ten-year old needles.

Pb showed a pattern similar to that of Sb for sites inside the city, but the association with traffic was weak or absent at the sites near major roads outside the city, likely related to large amounts of legacy deposition of Pb. More extensive studies should be undertaken to understand the distribution of the highly toxic Sb and Pb in the urban landscape as well as the exposure of humans and other organisms of the urban ecosystems.

## CRediT author statement

Håkan Pleijel: Funding acquisition, Conceptualization, Investigation, Writing - Original draft preparation. Jenny Klingberg: Conceptualization, Investigation, Formal analysis, Writing - Reviewing and Editing. Bo Strandberg: Investigation, Methodology (PAH analysis), Writing - Reviewing and Editing. Henrik Sjöman: Investigation, Writing - Reviewing and Editing. Göran Wallin: Conceptualization, Resources, Writing - Reviewing and Editing.

## Funding statement

This work was supported by the Swedish reserach council Formas (grant number 2017-00696).

## Declaration of competing interest

The authors declare that they have no known competing financial interests or personal relationships that could have appeared to influence the work reported in this paper.
