# Supplementary material for: Accumulation of antimony and lead in leaves and needles of trees: The role of traffic emissions
Source: Heliyon. 2023 Feb 8;9(2):e13548. doi: 10.1016/j.heliyon.2023.e13548 (PMC9947302; doi:10.1016/j.heliyon.2023.e13548)
Supplement: Multimedia component 2 [file mmc2.docx]

**PAH analysis**

## **Chemicals and Reagents**

All adsorbents, silica gel 60, aluminium oxide 90 active neutral and sodium sulfate (Merck, Darmstadt, Germany) were cleaned by thermal treatment at 450°C and activated at 100°C before use. All solvents were of glass distilled quality (Merck, Darmstadt, Germany). A deuterated internal standard (IS) mixture (1 ng µL^-1^) containing the 16 U.S. Environmental Protection Agency (US-EPA) priority PAHs (Dr. Ehrenstorfer (Augsburg, Germany) were used. Three native mixtures of, all at 1 ng L^-1^, containing 18 parent PAHs and among them the 16 US EPA PAHs (Dr. Ehrenstorfer, Augsburg, Germany), 14 alkylated species (Ultra Scientific, North Kingstown, RI, USA) and six dibenzothiophenes (DBTs) (Toronto Research Chemicals, Toronto, Ontario, Canada), were used for detection and quantification of target compounds. Octachlorornaphthalene (OCN) (Ultra Scientific, North Kingstown, RI, USA) (1 ng L^-1^) was used as recovery standard (RS).

**PAH air concentration measurement**

The PUF (polyurethane foam) disk samplers (14 cm diameter, 1.2 cm thickness, surface area 360 cm^2^, density 0.035 g cm^−3^, Klaus Ziemer GmbH, Germany), used for air PAH sampling, were housed in two stainless steel domes (Tisch Environmental, Inc., OH, USA). This sampler design has been calibrated for a number of PAHs, both gaseous and particulate-associated compounds (Harner et al., 2013; Bohlin et al., 2014). Prior to deployment, the PUF disks were precleaned by Soxhlet extraction for 24 h using dichloromethane (DCM), dried under vacuum, and stored in multiple layers of solvent-rinsed aluminium foil inside airtight polyethylene zip bags. After air sampling, the PUF disks were enveloped in rinsed aluminium foil, placed in air-tight polyethylene zip bags, brought to the laboratory, and stored at -18°C until extraction.

The published uptake rates by Bohlin et al. (2014) were used to quantify air concentrations of all 15 of the US EPA PAHs except naphtalene. Two-ring PAHs, such as naphthalene, may after a 28-day sampling period be in the curvilinear phase of uptake or have reached saturation in the sampling material (Bohlin et al., 2014). Thus, accurate quantification of this compound could not be made. The alkylated PAHs and DBTs were quantified using published uptake rates by Harner et al. (2013) or using the uptake rates for a corresponding, or closely related, US EPA PAH compound. In total, the concentrations of 32 PAHs were determined, and among them the 15 US EPA priority PAHs and 14 alkylated species, as well as 6 DBTs.

**Sample extraction, clean-up and analysis**

The extraction equipment used was a Dionex ASE 350 Accelerated Solvent Extractor equipment (Thermo Fisher Scientific, Inc. MA, USA). PUF samples were placed in extraction cells of 60 mL, filled with 4 g of silica in the bottom. Samples were spiked with 40 μL of the IS mixture and then extracted using dichloromethane as solvent. The extraction was performed at 100°C and three static time cycles. Following extraction, all samples were evaporated and transferred to 10 mL amber vials. The samples were then concentrated under nitrogen flow until only a third of the initial volumes was left, and solvent exchanged using n-hexane (ca 3 mL), and finally evaporated to ca 1 mL. Target compounds were separated on an Agilent 5975C mass spectrometer (MS) coupled to a 7890A gas chromatograph (GC, Agilent Technologies). Samples (2 μL) were injected using an Agilent autosampler unit. The capillary column used was a DB-5MS (30 m × 0.25 mm, 0.25 μm, Agilent Technologies). Helium was the carrier gas at a flow rate of 1.0 mL/min. The temperature program was as follows: initial temperature 50°C for 3 min; ramp at 10°C/min to 180°C and held for 5 min; ramp at 3°C/min to 300°C and held for 20 min; injection at oven temperature at 250°C, and transfer line at 250°C. Electron impact ionization (EI) was performed at 70 eV energy and at a 230°C ion source temperature. The method is assured with native mixtures of all target compounds as given above.

**Quality assurance**

Consistent recoveries (40-110%) were obtained for all IS compounds that were added to and used for correction of PUF samples. Field PUF blanks were included in the field sampling but were allowed to remain unopened in the bag. The field and laboratory PUF blanks were analyzed in parallel with the PUF samples. Minor residues only of some 2-4 ringed parent PAHs occurred, although the amount occurring was <10% of the amount found in the samples. All results were corrected for the blanks, respectively. The limits of detection (LOD) were calculated as three times the standard deviation of the values for the blanks or the background noise of these blanks. One standard reference material (SRM 1649b) was used as quality control (QC). The measured levels lie, for most part, within 30% of the certified levels (Figure S2).


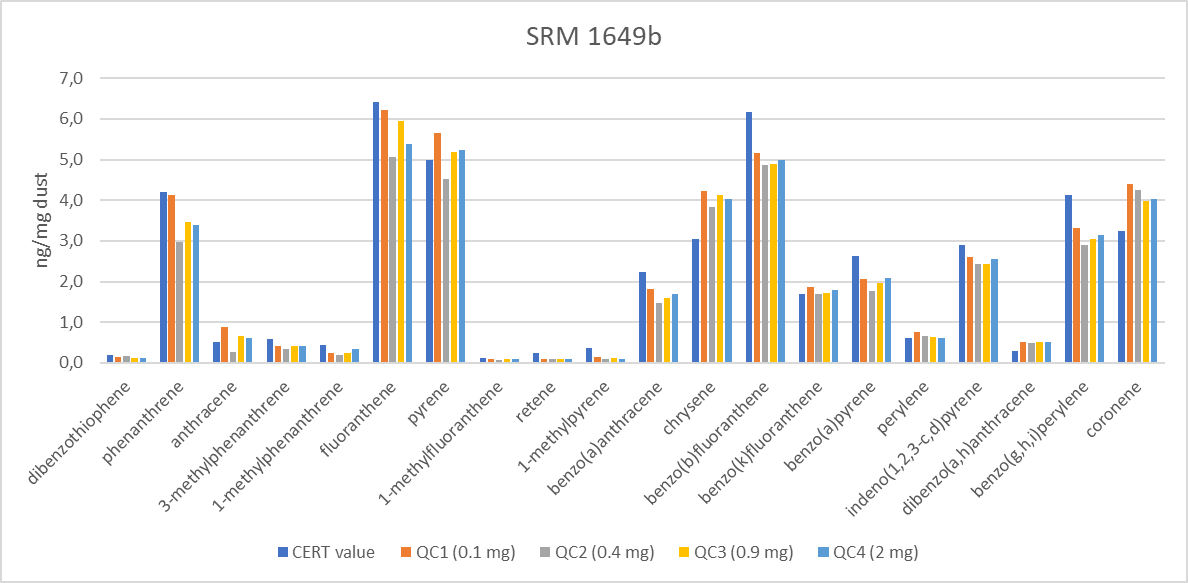


Figure S2. Results of four quality control (QC) samples (NIST SRM 1649b) compared to published certified values of 20 polycyclic aromatic compounds. The QC samples comprise weighed masses 0.1 - 2 mg dust.
